# The Medical Schools of London

**Published:** 1895-09-07

**Authors:** 


### The Medical Schools of London.

### MEDICAL STUDY IN LONDON.

To one who has decided to adopt medicine as a profession, the choice of a medical school in which to obtain the necessary education presents, and must present, considerable difficulties.

Individual desires and idiosyncrasies will turn different men to different centres of education. Some will prefer London, some the provinces, some Scotland, others Ireland; and among each group there will be those who will choose, or who, by circumstances, will be driven to this, that, or the other school of medicine. There can, however, be but little doubt that to the man who is free and unfettered in his choice, and is ready and willing to take trouble and to lay out his time to the best advantage, London offers a field for study such as is possessed by no other place in the kingdom.

No doubt, for those who take the view that life is short, and that the brain at the best is only capable of absorbing a certain amount of knowledge in the time relegated to study, there will be apparent advantages in attaching themselves to schools such, for example, as certain of the Scotch Universities, in which the art of teaching has been profoundly studied, and where the daily course which shall eventuate in an M.D. is laid down with great exactitude. But, for broader-minded men, we have no doubt of the advantages of London, or that, if once the University were brought in touch with the wants of the medical profession, so that a degree in medicine could be attained with the same facility here as it is in Scotland, London should stand head and shoulders above all other places as a centre for medical study for English-speaking people. We shall, then, confine ourselves to a description of the various medical schools of London, and, taking them seriatim, shall describe some of their individual advantages and peculiarities. We would premise, however, that, much as they may differ in some respects, there is no medical school in London which has not on its staff men who are well known—nay, celebrated—in their own departments and men who are thoroughly well competent as teachers. On the other hand, there is probably no school in London which has not one or more "duffers" in its teaching staff, men who, however deep their knowledge, have failed to learn the art of conveying it to others. There is then no one school which is all good, any more than there is any one which is all bad or even indifferent. The variety between the different schools cannot be expressed in such terms. One is strong on one side, one on another. One has a reputation for the clinical teaching in its wards, another for the systematic teaching in its class-rooms. One may have the best dissecting-room, another the best laboratory, and so on. Then some schools are more expensive than others, and it is probably very often true that the more expensive schools are frequented by students who, having more money than others, have more expensive tastes, and thus it may well occur that the general tone, the average standard of living, of dress, and of amusements, at one school may be of a more expensive type than at another; and although this is an influence of which a student with any backbone may, if he chooses, shake himself free, it is a matter not to be entirely ignored in the choice of a place of study for a youth of a sensitive disposition, but of a slender purse. On the other hand, it must not be forgotten that some schools are much better provided with prizes and with scholarships than others are, and although we would much deprecate the plan of arranging a student's finances on the very slender basis of his being able materially to assist himself by gaining scholarships, still, the extent of the provision of such aids to comfortable subsistence offered by the different colleges is a point to be carefully considered by those whose brains are of that peculiar type which enables their owners to glide easily through the strange pitfalls of the examination-room. We hesitate to say that the prizes are to the strong, or that the student who, term after term, is decorated with medals and carries off arms full of books is of necessity the best man or will make the best practitioner; but certainly some men and boys have the knack of so formulating and presenting their knowledge as to pass examinations with comparative ease, and to such the number and value of scholarships at a given school is a matter of no small importance, while to the lad who after all his schooling can only just scrape through his entrance examination such prizes are a matter of indifference, they are too far beyond him for consideration. The question then is not, which is the best medical school in London? but, which is the most suitable in the particular case? We have, therefore, on the following pages put together certain information which we hope will be read with interest and will prove of some assistance in solving this knotty point. It will be observed that there are considerable differences between the different schools both in fees, in equipment, and in strength of staff, and these we leave to the readers' judgment, refraining from making those comparisons which are proverbially odious. But for the comfort of parents and guardians, and for the stimulation and encouragement of students, who are sadly apt, so soon as they are fixed at one place, to raise loud lamentations that they had not been sent to somewhere else, we would say that we are quite certain that at the present time there is no medical school in London at which an intelligent and careful student may not qualify himself to pass his examinations with credit and to practise his profession with distinction.

Not including the London School of Medicine for Women, there are in London eleven hospitals which have medical schools. These eleven schools may be conveniently divided into three classes. There are, in the first place, the three great endowed hospitals, St. Bartholomew's, Guy's, and St. Thomas's, each of which has its school attached. Then there are in London two completely equipped colleges — King's College and University College. Each of these has professors in many branches of knowledge, science, art, language, history, engineering, &c., as well as a complete medical department, and attached to each is a hospital for clinical teaching. Theoretically, these colleges ought to offer many advantages over mere schools connected with hospitals. One might expect that with their large number of students and their wide variety of studies, their museums and libraries, concerned with a multitude of interests outside the field of medicine pure and simple, and with the necessary commingling of students with many aims and varied ambitions, an education of much wider scope, a culture of much broader sympathies might be attainable than by mixing always with those who are engaged in the same studies, and are running along the same narrow track which, without side issues or collateral interests, is laid down as the shortest route from entrance examination to diploma. We fear that in practice this is not so, and that at the colleges as at the schools the medical students keep together and take but little interest in what lies outside the exact curriculum laid down by the Examination Board. It is a pity, and we think that every effort should be made to render these colleges something better than an accidental conglomeration of many class-rooms under one roof.

Then come the six medical schools, which neither form part of colleges, nor are connected with endowed hospitals. They are, one may say in every case, the direct outcome of the energy and the fame of the medical staffs of the hospitals to which they are attached, and although by accident they come last upon our list, they are in no way inferior to the others, some of them being second to none in their teaching power, and all of them being cram full of clinical material. We shall now consider the individual schools, taking them in alphabetical order in each class.

### St. Bartholomew's.

The hospital of St. Bartholomew was founded in 1123 A.D., and there are records of a medical school being connected with the hospital as early as 1662. While, then, St. Bartholomew's is the oldest hospital in London, the medical school attached to it is the oldest metropolitan school. The hospital is still the largest but one in London, containing 744 beds, and it is the wealthiest of all. These facts imply much with regard to the status of the school. Age, coupled as it is in this case with continued usefulness, implies prestige; it has an exceptionally large staff of teachers, and the money that has been freely expended has provided every appliance which a fully-equipped medical school needs, not to speak of the generous benefactions which have provided very many prizes and scholarships open for competition at every stage of the student's career. In this latter respect St. Bartholomew's is exceptionally rich. The school buildings are comparatively new, having been completed in 1881 at a cost of £50,000. They include an admirably fitted-up set of laboratories, and a dissecting-room, which is one of the finest, if not the finest, in London. The museums also are a special feature, the pathological museum being one of the finest of its class in the world. Needless to say that St. Bartholomew's is one of the "large" schools. But it is also one of the largest hospitals, and the appointments are numerous enough to give every student work. Moreover, there are so many teachers that, large as is the number of students, those classes in which individual teaching is required are not proportionally large. The fees at St. Bartholomew's are higher than at any other school in London, the composition fee being 150 guineas, and one cannot help feeling that, unless public opinion and general repute is to be looked on as a mere matter of caprice, there must be some special attractiveness about this great old school to keep its classes so well filled while its fees remain so high.

Attached to the school is a residential college, but the accommodation in it is limited. Dr. Shore, the warden, keeps a list of approved lodgings, as to which he has made careful inquiries, and in the case of young men coming up to London for the first time he strongly urges that this list should be consulted.

Connected with the hospital and school there are various societies, one of which—the Abernethian—has historical associations of a most interesting kind.

The various clubs have been amalgamated, and the members have the advantage of the use of the Abernethian reading-room, the smoking-room, and of a permanent recreation ground of their own at Winchmore Hill, one of the best and prettiest athletic grounds in the neighbourhood of London.

### Guy's.

Within a few minutes' walk of London Bridge Station is one of the best known of the hospitals of London, one which, by its short and distinctive name of Guy's, has become familiar to the British public as being a favourite *locale* with English novelists from Dickens onwards.

Guy's Hospital, again, is one of the large, if not the largest, of the medical schools of London, and by the energy of those engaged in its management its prestige increases year by year. That the school retains the good name which it has now held for many generations as a sound and practical teaching centre may be judged of by a remark in the Dean's report, in which he states that, notwithstanding the prevailing opinion that the profession is overcrowded, it has not been found, in connection with the efforts made in this school to obtain for qualified students remunerative occupations on the completion of their hospital course, that there is any increasing difficulty in obtaining such posts. This would seem to indicate that there is still a demand for well-qualified practitioners, and that a Guy's education is looked upon as at least presumptive evidence of qualification for responsible work. The results of the examinations have pointed in the same directions, for the percentages of successes at the various examinations of the Conjoint Board achieved by students of this hospital have in some subjects very considerably exceeded, and in all but one subject proved better than the general average for all schools. In the honours examination for the M.B. degree at the London University more places were awarded to Guy's men than to students from any other medical school, nearly one-fourth of the number of honours falling to Guy's men; and when it is stated that thirteen Guy's men presented themselves for the M.D., and twelve for the B.S. examination, and that in neither case did any of them fail to satisfy the examiners, the efficiency of the Guy's school is, we think, demonstrated beyond the possibility of cavil.

The hospital contains accommodation for 695 patients, the beds in constant occupation numbering rather more than 500.

Attached to the hospital is a very large lying-in charity, the largest in fact which is attached to any English school of medicine, and seeing the important part which a complete knowledge of the obstetric art plays in general practice, it is not impossible that the large opportunities which fall in the way of Guy's students in this field may have much to do with their after success. The number of cases attended by the extern obstetric assistants last year was 3,110.

Guy's Hospital also has a complete dental school which affords the students all the instruction required for a licence in dental surgery. This gives to the general students not only an opportunity of acquiring such knowledge of dental surgery as is required for the ordinary diploma, but gives them exceptional facilities for obtaining a practical experience in the administration of anæsthetics. Attached to the hospital is a collegiate residence for students, in which about 60 men reside under the supervision of a resident warden. In the same building also, which is connected with the hospital by a subway, accommodation is given for the students' club. Considering the accommodation given in the college, we look upon the rents charged for the rooms as being cheap, and during the second part of the curriculum, when it is of great importance to be actually in the midst of the hospital work, this residence must be of the extremest advantage to the students. During the first two years of student life, however, when the work is not hospital work at all, there is much to be said in favour of sleeping in the suburbs, especially when the school has such railway facilities as in the case of Guy's. We must not omit to say that Guy's is well provided with societies and clubs. The Clubs Union Grounds at Honor Oak Park, nine acres in extent, can be reached in less than twenty-five minutes by rail, and among the societies the Guy's Physical Society still maintains its old position. The composition fee at Guy's is £150.

### St. Thomas's.

Standing as it does, facing the Houses of Parliament on the opposite side of the river, and forming part, and a very striking part, of a view which is looked upon with interest by every visitor to the metropolis, the external architectural features of St. Thomas's Hospital are probably better known than those of any hospital in London. A modern hospital, erected in a magnificent situation, having behind it the vast population whom it serves, and in front the open river and, beyond the splendid buildings of Westminster, the parks of the West-end, built also at a lavish expenditure of money, and with every effort to give light and air to all its inmates, St. Thomas's Hospital is at present the most striking example which London presents of what a hospital can be made. Structurally it is one of the finest in the world, and the student spending his time in its noble wards cannot fail to have fixed in his mind an impression of the importance and the efficacy of good sanitary conditions which will remain fixed throughout his life. The school is worthy of the hospital, and since the opening of the recent additions, consisting of laboratories and the students' club, it has had ample accommodation for all purposes of medical education. St. Thomas's gives special attention to the scientific side of medical study, and the results at the examinations show that the instruction provided in the subjects of the London University examination and others is excellent. The opportunities for clinical work also are unsurpassed. The composition fee is £150.

### King's College.

King's is a college having many departments, and among them a complete faculty of medicine which is housed in the well known-buildings in the Strand adjoining, and on the river side continuing the frontage of, Somerset House. The hospital is in Portugal Street, Lincoln's Inn Fields, a distance of five or ten minutes' walk. King's College is in a special way identified with the Church of England, and all its teachers, in medicine as in other faculties, are members of the Church. Rather than make any alteration in this, which has been held to be one of the essential features of its constitution, the college authorities has recently submitted to the deprivation of a considerable Government grant which was held not to be available for institutions demanding any religious test. Regarding the wisdom of this course we wish to make no remark, but it behoves parents and guardians to know that this college alone, among medical schools in London, has ecclesiastical relations, and that among the lectures which are compulsory in the first year are those in Divinity. There is in the college a handsome chapel in which divine service is regularly celebrated, and it will be noticed by the visitor that matriculated students are expected to wear the college cap and gown. Rooms are provided within the walls of the college for a limited number of matriculated students, who are subject to the same general rules as undergraduates at the Universities; and in regard to this it is to be observed that the rent of these rooms includes the dinner. Differing again from other schools, the Dean of the Medical Faculty of King's College at the end of each term prepares a report of the attendance, general character, and conduct of each student, which is forwarded to his parent or guardian, after being submitted to and signed by the principal. It would appear, then, as if at King's the student was expected to live somewhat more according to rule, and to remain somewhat more *in statu pupilaris*, than at other colleges, and no doubt this will meet with much approbation in the minds of many parents, although the practical efficacy of such measures may perhaps be doubted.

Both in regard to appliances, staff, and clinical opportunities the students at King's College have every advantage. Although the hospital is very much smaller than any of those we have hitherto spoken of, it has had on its staff a brilliant succession of teachers, and is known to all the world as the place in which for so many years Sir Joseph Lister enforced the principles and practice of antiseptic surgery.

King's College has done much for the scientific side of medicine, and of recent years has arranged a very complete organisation for the practical teaching of bacteriology in laboratories specially arranged for the purpose. Special attention also is given to hygiene and public health, in which department a full course is provided.

There is a medical society in connection with the college, and a recreation ground close to Wormwood Scrubbs Station for the use of the college and school, and an athletic club has been formed for its management. The composition fee is £135.

### University College.

The faculty of medicine at University College has for long past included a series of brilliant teachers and of men of note in the various branches of medical knowledge, and it is in no way surprising that a school with such a long list of distinguished professors should have obtained a reputation for thoroughness, and that tradition should attach to the men whom it sends forth a character for scientific learning.

The college buildings are extensive, and give every opportunity for study and research; and it may here be noted that University College is more than a mere teaching school, for in the laboratories of some of its professors it gives a home to many original investigations and much deep research, and have been the birthplace of many additions to our knowledge. University College may well pride herself on the success with which she has devoted herself to higher scientific work, and it is stated that, on a rough estimate, two-thirds of her men prepare for the London University degrees, and that the highest honours of the London examinations have been carried off by this school oftener than by any other. This speaks well, not only for the character of the teaching, but for that of the students, who would hardly have such success did they not enter earnestly into their work.

The college, however, has for long laboured under the disadvantage of being connected with a hospital which is small and old-fashioned in construction. This, however, is about to be remedied very shortly. The land behind the present hospital has been acquired, and it is hoped that on it the erection of new buildings will soon be commenced.

Nevertheless, small as the hospital is, extremely good work is done there, and the students have the advantage of dealing with cases which are to a large extent specially selected for the purposes of clinical teaching.

There is a large out-patient department, of which full use is made, and all the special departments are thoroughly organised for teaching purposes.

Attached to the hospital there is also a large maternity department, in connection with which 2,447 midwifery cases were attended last year.

All the offices which are open to the students are free, and the house surgeons, house physicians, and obstetric assistants receive free board and lodging. The composition fee is 135 guineas.

### Charing Cross Hospital.

At Charing Cross, which many people look upon as the very centre of London, land is valuable, and space for hospital and school is hard to get. Nevertheless, in this busy centre of an ever-moving crowd the existence of a hospital, a refuge to which those may be taken who fall in the race, is an absolute necessity, and we all know how necessary to the action and effective working of a hospital is a medical school, and although the Charing Cross Hospital and School may be looked upon as among the smaller of their kind, they none the less fill an important place in the economy of London life.

The school buildings adjoin the hospital, being connected with it by a subway, and are conveniently arranged. Every requisite for a medical school has found a place, and excellent teachers give instruction in all the subjects of medical study. With surgical cases in particular Charing Cross is always well provided. At the adjoining Royal Westminster Ophthalmic Hospital clinical instruction in eye diseases is given daily. Nothing is more difficult than for the authorities of a medical school in such a place as London to exercise supervision over the conduct of their students outside the hospital or the school; but at Charing Cross it is one of the rules of the school that conduct on the part of a student outside the hospital or school which is calculated to bring discredit on either renders the student liable to suspension or expulsion, and no doubt this will exercise a restraining influence over many who might otherwise be tempted to act foolishly. The composition fee at Charing Cross is £115 10s., which is reduced to 100 guineas in the case of sons of medical men.

### St. George's.

With a splendid frontage to Hyde Park Corner, looking on to the daily meeting-ground of the most wealthy and pleasure-seeking section of society, St. George's Hospital ministers to the needs of a vast industrial population in the south-west of London and to a large number of servants who, however comfortably situated while health continues, are too often both friendless and far from home when sickness overtakes them. The building occupies a singularly favoured site in the midst of the western residential quarter at the junction of Hyde Park with the Green Park, where it is environed by open spaces. It has recently undergone reconstruction, and now contains 350 beds, its virtual capacity being further increased by the acquirement of a large convalescent establishment of 100 beds in the neighbourhood of Wimbledon, to which convalescing patients are drafted as soon as they can bear removal.

Unusual opportunities are offered at St. George's for clinical study, as, contrary to the practice at many hospitals, the wards are open to the students at all convenient hours with only nominal restrictions. The medical school block is well planned and commodious, and in it are to be found a well-stocked library, a reading-room, and a refreshment-room. The hospital and school have many associations with the honoured name of John Hunter. It was he who founded the medical school; it was in one of the rooms of the hospital that he died; and in the museum severali nteresting relics of Hunter are preserved.

St. George's offers a good all-round medical education, but is especially strong on the clinical side. A good many university men finish their course at St. George's, having in many instances taken out the first half of their studies, the anatomy, physiology, &c., at Cambridge; in fact, special arrangements are made in regard to fees to suit the convenience of those who have passed the first or second M.B. examination at the older universities.

The arrangements for clinical instruction are very complete, and for those who are fortunate enough to be able to become house officers, as they are termed, a very convenient arrangement is made by which they are enabled to go straight forward from one appointment to another for twelve months, and if they become resident house officers, for another twelve months, and during the latter time, they reside and board in the hospital free of all expense.

Whatever be the reason—be it the site, or the nearness of the hospital to the better quarters of the town, or the tradition that it is in some way related to the Universities—it seems to be the fact that St. George's is much resorted to by the sons of wealthy men. As the result, perhaps, of tradition also, a good many of its students seem to aspire to the army and navy medical service. St. George's has no residential college. A list of approved lodgings, however, is kept, and of houses of medical men and others where St. George's students are received as boarders. The composition fee is £145, with certain additions for science teaching, chemistry, and pharmacy.

### The London Hospital.

This is the largest hospital not only in London, but in Great Britain, containing 786 beds, and its position in the neighbourhood of the extensive docks, factories, and workshops of the East of London renders it one of the largest accident hospitals in the world. It presents, then, a splendid field for clinical experience and for obtaining a practical acquaintance with all phases of disease. The special departments are well organised, and there is a large maternity charity giving ample opportunity for gaining experience in obstetrics.

The steady growth of the medical school in connection with this hospital may be traced in the numerous additions and improvements which have been made from time to time in the buildings devoted to its use. The present medical college, which was opened by the Prince and Princess of Wales in 1887, is a separate building, well arranged, providing good accommodation, and thoroughly fitted for its purpose. It contains all the necessary laboratories, dissecting rooms, lecture theatres, &c., together with a library and anatomical and pathological museums. With so large a hospital and such a multitude of patients it naturally follows that there is a large staff, and, what is of equal importance to the student, that there are a very large number of appointments, all of which are made without fee of any kind, all resident officers being provided with free board and room. There can be no doubt that students at the London Hospital have every opportunity, not only of getting an admirable medical education, both as regards teaching and such help as is to be gained by being attached to a thoroughly well-organised school, but also in the admirable opportunities which they have of gaining a practical touch with every form of disease.

There is no residential college in connection with the London, but there is a students' club which, generously aided as it has been by the members of the House Committee and the staff, has been able to make provision in the new buildings for students obtaining a comfortable meal at a reasonable cost and with little loss of time.

Nor are athletics neglected, and the Clubs Union have their own ground at Lower Edmonton, with an excellent pavilion, as well as a fives court in the hospital grounds open to all students, and lawn tennis courts in the hospital field open to members of the Clubs Union.

The neighbourhood of the London Hospital is much more open than would be imagined by many who associate all sorts of terrors with the name Whitechapel, and, while inexpensive lodgings can be obtained in the neighbourhood, really country suburbs are easily accessible by rail. The composition fee is 120 guineas, and a reduction is allowed to the sons of medical men.

### St. Mary's.

The medical school in connection with St. Mary's Hospital has made great strides in public favour, and has considerably advanced in importance during recent years. The provision for the teaching of the sciences is very good, and special attention has been given to the departments of bacteriology and public health. The hospital is within a few minutes' walk of the Great Western terminus, and serves a large and constantly growing district in the west of London. It contains 281 beds, and the Clarence Memorial Wing which is now being built will add largely to the accommodation, besides greatly increasing the capacity of the out-patient department.

Besides the more general teaching of medicine and surgery, great attention is paid to the special departments, and when the new wing is finished the arrangements for students obtaining a practical acquaintance with the diseases of the special organs will be very complete.

Quite a new departure also is about to be made in regard to the maternity department, it being intended to have lying-in wards in the hospital. This is one of the results of the careful use of antiseptics, it now being found to be safe and justifiable to do what formerly was quite impossible. St. Mary's will thus be the first of the metropolitan general hospitals to establish lying-in wards, and it is easy to understand of what importance these wards will be to the students in connection with the practical study of obstetrics.

The school has a small residential college in Westbourne Terrace, where students are boarded at the rate of £75 for the academic year. The fact that the Great Western Railway terminus is so near at hand makes it very easy to get into comparative country, Ealing and other places of the sort being very quickly reached.

There is a students' club situated within the hospital, with reading, smoking, and dining rooms. A medical society also is attached to the school, and there are various athletic clubs. The subscriptions to the library, the Medical Society, the Students' Club, and all the athletic clubs are included in the composition fee. The composition fee is £138.

### Middlesex Hospital.

This hospital, although actually situated in Mortimer Street, would by most Londoners be looked upon as forming the end of Berners Street. The hospital, which within the last two years has been renovated throughout, now contains 320 beds. The great increase in the number of surgical operations has necessitated the erection of a new operating theatre, in the construction of which every attention has been paid to all the requirements of modern aseptic and antiseptic methods. It is a model of its kind, and as such has recently been described in the pages of The Hospital.

A peculiarity of the Middlesex Hospital is that, under a special endowment founded in the year 1792, there are special wards for cancer, in which, if necessary, the cases can be kept under observation for a much longer period than is common in other hospitals.

The school, which is in a separate building although within the hospital grounds, is in a flourishing condition. The entire course of study required by the Conjoint Examining Board is fully provided, but for students who have not passed the Preliminary Scientific M.B. Examination of the University of London before entering, and who require instruction in the subjects for that examination, an arrangement is made by which they can attend the necessary classes at University College.

There is a large out-patient department connected with the hospital, and also a maternity department, which provides for the practical study of midwifery. Full provision is made for all the special departments of practice, and everything is done to make the school as perfect as possible.

Adjoining the new school buildings is the residential college, which provides for the board and residence of about thirty students, the chaplain of the hospital being the resident warden. The charges for the rooms vary from eight to ten guineas per term.

The composition fee is 120 guineas, but if a fee of 140 guineas is paid, the necessary instruction for the Preliminary Scientific M.B. Examination of the University of London can be obtained at University College without further fee.

### Westminster Hospital.

This is a very old charity, having come into existence at a time when there were no hospitals in London except St. Bartholomew's and St. Thomas's, for Guy's Hospital was not established until a few years later. Westminster Hospital is remarkable, in fact, as being the first in London which was founded by the voluntary contributions of the public. The present hospital building, however, was not occupied until 1834, and the new school buildings were opened in 1885.

The hospital now contains upwards of 200 beds, and provision is made for all the separate special departments into which both the study and the practice of medicine is now divided.

The Westminster School, so far as buildings are concerned, is, no doubt, one of the smaller of the London schools; nevertheless, the space has been made the most of, and there is no essential feature in which they come short. And, for some students, it may be safely admitted that a small school is the best. For one thing, it certainly ensures more intimate personal relations between the teacher and the student. Great as are the advantages of a large school in some directions, it is a necessity of the case that a large share of the actual supervision of the students falls to the demonstrator instead of the teacher himself. The conditions, however, of such a school as the Westminster allow of the teacher doing work which is at other schools done by proxy. The clinical teaching and the opportunities of clinical observation are admittedly good at Westminster, and it is beyond dispute that a thorough and complete medical education is there provided along with careful personal supervision. The composition fee is £115.

### School of Medicine for Women.

London is provided with one complete school of medicine at which women can go through the whole course of study required for qualification. It is situated in Handel Street, Brunswick Square, and has now been in existence for 21 years. Having been opened in 1874, it this year attains its majority, on which fact we may fairly congratulate those pioneers who, in the face of much opposition openly expressed, and much prejudice which was difficult to fight against, have succeeded in establishing both the right and the capacity of women for the profession of medicine. The first three years of the history of this school were years of struggle; but the worst was over when it had been recognised by one of the licensing bodies—the King and Queen's College of Physicians, Dublin—and when in 1877 lady students were admitted to the practice of the Royal Free Hospital, Gray's Inn Road. Since 1877 progress has been uninterrupted.

The course of education is the same for women as for men, they both pass the same examinations and both obtain the same diplomas. Although in the provinces there is a fair choice of qualifications open to women, only the London University and the Apothecaries' Hall in London at present open their doors to them.

The clinical work in connection with the London School of Medicine for Women is obtained at the Royal Free Hospital, which contains 160 beds, where every student is able to obtain ample experience in clerking and dressing.

The New Hospital for Women contains 42 beds, and is entirely worked by women, and some very good work is being done there. Many other responsible medical appointments have also recently been given to women doctors. The composition fee of the London School of Medicine for Women is £125.

### Cooke's School of Anatomy, Physiology, and Surgery.

In addition to the complete schools of medicine above described there is another which deserves a place in any account of medical education in London. It is the School of Anatomy, Physiology, and Surgery that is conducted by Mr. Thomas Cooke, F.R.C.S., assistant surgeon to the Westminster Hospital. Mr. Cooke's classes have been recognised by the University of London and other examining bodies. They are designed to meet the needs of (1) a limited number of "earnest" men who desire a thorough grounding in practical anatomy and experimental physiology; (2) of gentlemen who have been rejected at their anatomical and physiological examinations—these can get "signed up" from this school for the three or six months' supplementary work that they are now required to put in before re-examination; and (3) of qualified men who wish to obtain some of the higher qualifications, or to compete for appointments in the army, navy, and Indian medical services. Mr. Cooke is an enthusiastic teacher of large experience, whose work has been so good that it has been rewarded by recognition on the part of the examining bodies.

